# Comparison of Safety of Leadless Pacemakers and Transvenous Pacemakers: A Meta-Analysis

**DOI:** 10.7759/cureus.45086

**Published:** 2023-09-12

**Authors:** Mahesh Gangannapalle, Obinna Monday, Anurag Rawat, Ugonna A Nwoko, Arun Kumar Mandal, Maham Babur, Tayyaba J Khan, Sujith K Palleti

**Affiliations:** 1 Medicine, University of Perpetual Help System Dalta, Laspinas, PHL; 2 Medicine, Norfolk and Norwich University, Norwich, GBR; 3 Interventional Cardiology, Himalayan Institute of Medical Sciences, Dehradun, IND; 4 Medicine, American University of the Caribbean School of Medicine, Cupicoy, SXM; 5 Medicine, Manipal College of Medical Science, Pokhara, NPL; 6 Internal Medicine, Women Medical and Dental College, Abbottabad, PAK; 7 Medicine, Liaquat University of Medical and Health Sciences, Jamshoro, PAK; 8 Nephrology, Louisiana State University Health Sciences Center, Shreveport, USA

**Keywords:** meta-analysis, complications, safety, transvenous pacemakers, leadless pacemakers

## Abstract

Pacemakers have been accessible for six decades, and clearly defined criteria for pacemaker implantation have been established. Within the contemporary clinical practice, two dependable pacing platforms exist leadless pacemakers and transvenous pacemakers. The aim of this meta-analysis is to compare the safety of leadless pacemakers to transvenous pacemakers. This meta-analysis adhered to the guidelines outlined in the Preferred Reporting Items for Systematic Reviews and Meta-Analyses (PRISMA) 2020 framework. A comprehensive and systematic search was conducted across various databases including Scopus, Cochrane Library, and EMBASE, spanning from inception to August 15, 2023. The primary outcomes assessed in this meta-analysis were total complications, all-cause mortality, and device-related complications. Furthermore, secondary outcomes evaluated encompassed the need for reintervention, occurrences of pneumothorax, pericardial effusion, endocarditis, hemothorax, and hematoma. Total 17 studies were included in this meta-analysis. The findings of this study showed that patients with leadless pacemakers had a lower risk of total complications, device-related complications, pneumothorax, and endocarditis. The risk of reintervention was significantly lower in the leadless pacemaker group. However, compared to a transvenous pacemaker, the risk of pericardial effusion was significantly higher in the leadless pacemaker group. It is important to acknowledge the limitations arising from the lack of extensive long-term follow-up data for leadless pacemakers. As technology evolves, continued research will be essential in uncovering the full spectrum of prolonged complications associated with these devices.

## Introduction and background

Pacemakers have been accessible for six decades, and clearly defined criteria for pacemaker implantation have been established based on evidence from randomized controlled trials (RCTs) [1,2]. Within contemporary clinical practice, two dependable pacing platforms exist: leadless pacemakers and transvenous pacemakers [3]. Despite significant technological advancements in implantable electronic cardiac devices, the implantation procedure for conventional transvenous pacemakers - entailing a subcutaneous pulse generator and transvenous leads - has endured without alteration. The subcutaneous pocket and transvenous leads are the primary culprits behind complications arising from transvenous pacemaker implantation, affecting approximately 3%-12% of patients [4,5]. To mitigate the incidence of these issues, the concept of leadless pacemakers was introduced in the 1970s [6]. Leadless intracardiac pacemakers, deployed through a minimally invasive femoral vein approach, are fully implanted within the right ventricle and are currently utilized in patients requiring single-chamber ventricular pacing [7].

Approval for the use of leadless pacemakers by the United States Food and Drug Administration (FDA) was initially granted in April 2016 [8]. In recent times, leadless pacemakers have gained considerable popularity. These self-contained devices integrate a pulse generator, battery, and electrode into a single unit, addressing certain limitations of conventional transvenous pacemakers [9]. Positioned entirely within the right ventricle, initial clinical trials have demonstrated the excellent performance of leadless pacemakers in terms of device electrical parameters for up to a year. These early trials also highlighted lower rates of lead and pocket-related complications compared to traditional pacemakers, with reported rates ranging from 3.4% to 6.5% [9,10].

Notably, recent findings have suggested a potential safety advantage of leadless pacemakers over transvenous pacemakers, although these studies encompassed relatively small patient cohorts. As a result, this meta-analysis seeks to enhance precision by consolidating data from studies directly comparing leadless pacemakers to transvenous pacemakers, aiming to establish a robust evaluation of safety outcomes. The aim of this meta-analysis was to compare the safety of leadless pacemakers to transvenous pacemakers.

## Review

Methodology

This meta-analysis adhered to the guidelines outlined in the Preferred Reporting Items for Systematic Reviews and Meta-Analyses (PRISMA) 2020 framework.

Literature Search and Inclusion Criteria

A comprehensive and systematic search was conducted across various databases including Scopus, Cochrane Library, and EMBASE, spanning from inception to August 15, 2023. The search utilized specific key terms such as “leadless cardiac pacing,” “leadless pacing,” “Micra transcatheter pacing,” “leadless cardiac pacemaker,” and “leadless pacemaker,” in conjunction with terms like “standard pacemaker,” “permanent pacemaker,” “conventional pacemaker,” and “traditional pacemaker.” Additionally, a manual search of references in the included studies was conducted to identify potential supplementary research. The acquired records from electronic databases were imported into EndNote X9 software, and duplicate entries were eliminated. The screening process encompassed an initial evaluation of titles and abstracts, followed by a full-text examination to ascertain adherence to eligibility criteria.

Eligibility Criteria

This meta-analysis incorporated any RCT or non-RCT (non-RCT) that juxtaposed leadless pacemakers with transvenous pacemakers while reporting relevant outcomes. Inclusion was confined to studies published in the English language. Notably, meta-analyses, reviews, editorials, and expert opinions were excluded. The primary outcomes assessed included total complications, all-cause mortality, and device-related complications. Furthermore, secondary outcomes evaluated encompassed the need for reintervention, occurrences of pneumothorax, pericardial effusion, endocarditis, hemothorax, and hematoma.

Data Extraction and Quality Appraisal

Data extraction entailed gathering pertinent details from included studies, such as the first author, publication year, study design, geographic location, sample size, follow-up duration, participant characteristics, and outcomes. This process was independently conducted by two authors utilizing a pre-designed data extraction form in Excel. Any discrepancies that arose during data extraction were resolved through consensus. The quality of included studies was evaluated using the New Castle Ottawa Scale (NCOS), which assesses studies across categories such as group selection, group comparability, and exposure or outcome assessment. The cumulative score from these categories indicated the overall study quality.

Data Analysis

Data analysis was executed using RevMan Version 5.4.1 (The Cochrane Collaboration) and STATA 16.0 (Stata Corp). Risk ratios (RR) were presented alongside their corresponding 95% confidence intervals (CI). A significance threshold of P-value < 0.05 was employed. Heterogeneity was assessed through I-square values, where an I-square exceeding 50% was deemed indicative of significant heterogeneity. In cases of notable heterogeneity, RR calculations were performed using a random-effects model; conversely, a fixed-effects model was employed for analysis when heterogeneity was insignificant. We performed meta-regression to understand to understand the potential sources of heterogeneity observed across the included studies in our meta-analysis taking total complications (primary outcome) as a dependent variable. Meta-regression allows us to explore how various covariates or factors may influence the effect sizes or outcomes reported in the studies.

Results

**Figure 1:**
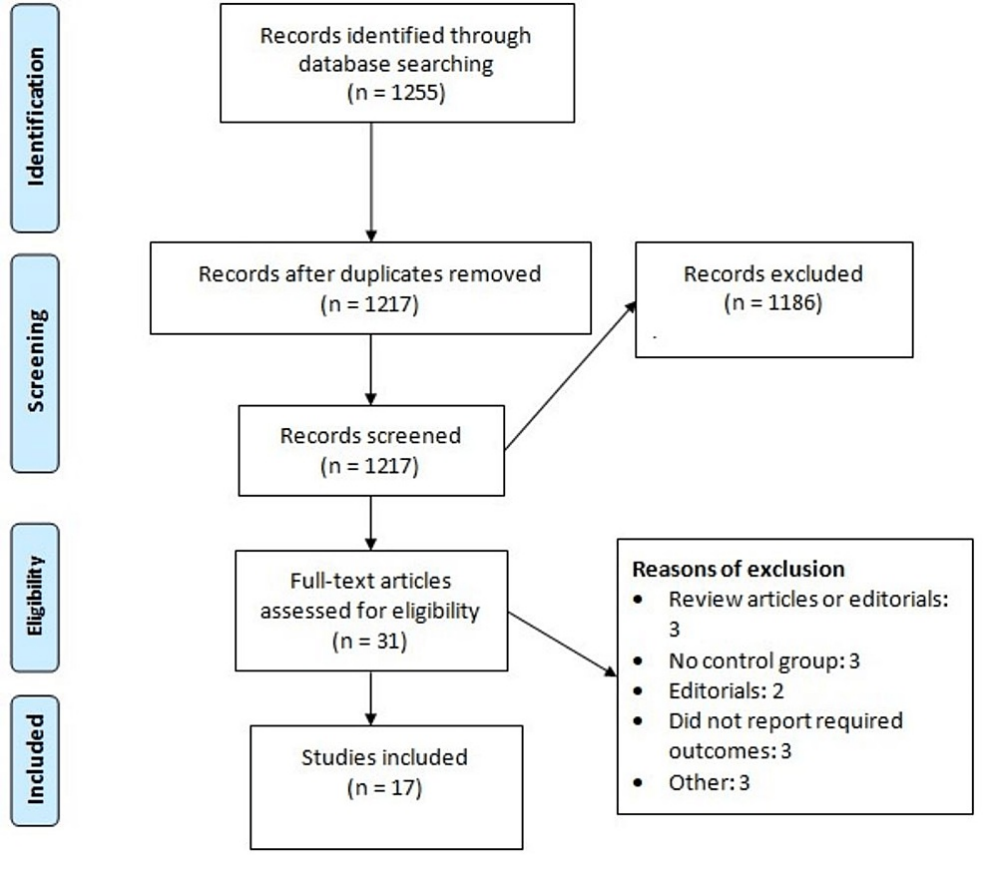
PRISMA flowchart showing the process of study selection

The online database search yielded 1,255 citations. After screening abstracts and titles, we excluded 1,186 studies. We obtained full text of 31 studies and detailed assessment was performed based on pre-defined inclusion and exclusion criteria. Finally, 20 studies were included in this meta-analysis. Figure 1 shows the PRISMA flowchart of study selection. Table 1 presents the characteristics of the included studies. Table 2 presents a quality assessment of the included studies. Three domains were assessed including selection, comparability and exposure, and outcome ascertainment. Each of these domains is further divided into specific criteria, and studies are awarded points for meeting these criteria.

**Table 1:** Characteristics of included studies RC: Retrospective cohort, PC: Prospective cohort

Author Name	Year	Region	Study Design	Groups	Sample Size	Mean Age (Years)	Males (%)
Bodin et al. [11]	2022	France	RC	Leadless	1344	70.7	57.9
				Tranvenous	1344	83.4	60.2
Boveda et al. [12]	2023	United States	RC	Leadless	5514	77.7	56.8
				Tranvenous	6422	80.6	59.4
Cantillon et al. [13]	2018	United States	RC	Leadless	718	75.6	62.3
				Tranvenous	1436	76.1	63
Chami et al. [14]	2022	United States	RC	Leadless	6219	79.5	55.9
				Tranvenous	10212	82	56.8
Martinez-Sande et al. [15]	2020	Spain	PC	Leadless	245	83.6	27.3
				Tranvenous	198	79.2	62.1
Moore et al. [16]	2019	United States	RC	Leadless	10	82.8	60
				Tranvenous	23	84	52
Okuyama et al. [17]	2020	Japan	RC	Leadless	10	86.5	20%
				Tranvenous	14	83	21
Pagan et al. [18]	2020	United States	RC	Leadless	183	89.5	51.9
				Tranvenous	119	89.9	40.3
Palmisano et al. [19]	2021	Italy	PC	Leadless	77	77.5	71.4
				Tranvenous	77	78.3	63.6
Palmisano et al. [20]	2023	Italy	RC	Leadless	442	73.2	68.3
				Tranvenous	442	74	68.8
Reynolds et al. [7]	2016	United States	PC	Leadless	725	75.9	58.8
				Tranvenous	2667	71.1	55.1
Sanchez et al. [21]	2020	United States	RC	Leadless	67	73	54
				Tranvenous	131	74	27
Sasaki et al. [22]	2022	Japan	RC	Leadless	58	81	38
				Tranvenous	58	82	40
Tachibana et al. [23]	2020	Japan	RC	Leadless	35	90.1	44.4
				Tranvenous	27	90.9	40
Vaibhav et al. [24]	2018	United States	RC	Leadless	90	80.5	63
				Tranvenous	90	78.2	63
Yarlagadda et al. [25]	2018	United States	RC	Leadless	60	74	48
				Tranvenous	67	74	24
Zucchelli et al. [3]	2021	Italy	RC	Leadless	100	77.5	77
				Tranvenous	100	78.8	67

**Table 2:** Quality assessment of included studies using New Castle Ottawa Scale Selection: Out of 3, Comparability: Out of 2, Exposure or outcome assessment: Out of 4

Author Name	Selection	Comparison	Outcome	Overall
Bodin et al. [11]	2	1	3	Fair
Boveda et al. [12]	3	2	4	Good
Cantillon et al. [13]	2	2	3	Good
Chami et al. [14]	2	2	3	Good
Martinez-Sande et al. [15]	2	2	4	Good
Moore et al. [16]	2	2	4	Good
Okuyama et al. [17]	3	1	3	Good
Pagan et al. [18]	3	2	3	Good
Palmisano et al. [19]	2	2	4	Good
Palmisano et al. [20]	3	2	3	Good
Reynolds et al. [7]	3	1	3	Good
Sanchez et al. [21]	3	2	3	Good
Sasaki et al. [22]	3	2	4	Good
Tachibana et al. [23]	2	1	3	Fair
Vaibhav et al. [24]	2	1	2	Fair
Yarlagadda et al. [25]	2	2	3	Good
Zucchelli et al. [3]	3	2	4	Good

Primary End Points

**Figure 2:**
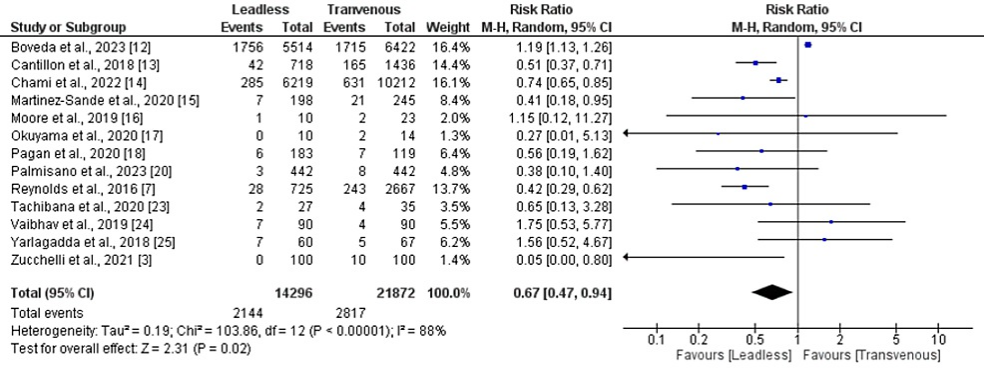
Comparison of total complications between leadless and transvenous pacemaker groups Sources: References [3,7,12-18,20,23-25]

*Total complications: *Thirteen studies compared the risk of total complications between leadless and transvenous pacemaker groups. As shown in Figure 2, the risk of all complications was significantly lower in the leadless pacemaker group compared to the transvenous pacemaker group (RR: 0.67, 95% CI: 0.47-0.94). Significant heterogeneity was reported among the study results.

**Figure 3:**
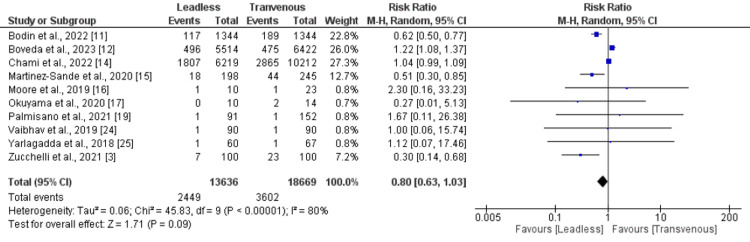
Comparison of mortality between leadless and transvenous pacemaker groups Sources: References [3,11,12,14-17,19,24,25]

*Mortality: *Ten studies were included in the pooled analysis of the risk of mortality. A pooled analysis of 10 studies showed that the risk of all-cause mortality was higher in the transvenous pacemaker group compared to the leadless pacemaker group. However, the difference was statistically insignificant (RR: 0.80, 95% CI: 0.63-1.03) as shown in Figure 3. Significant heterogeneity was reported among the study results.

**Figure 4:**
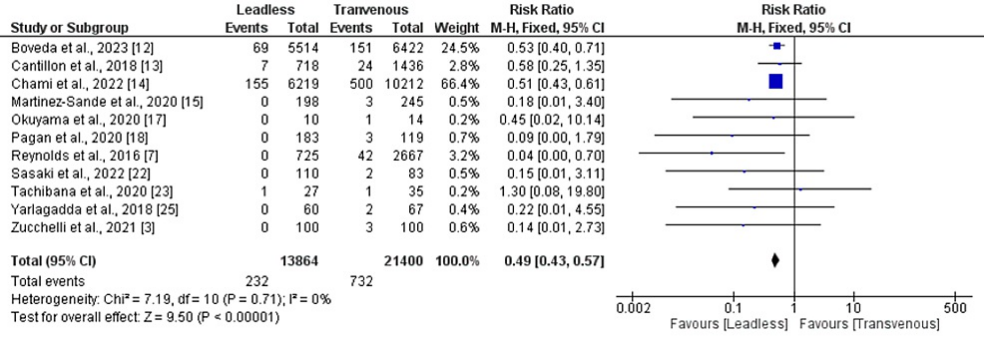
Comparison of total device-related complications between leadless and transvenous pacemaker groups Sources: References [3,7,12-15,17,18,22,23,25]

*Device-related complications: *Eleven studies were included in the pooled analysis of device-related complications. As shown in Figure 4, the risk of device-related complications was significantly higher in the leadless pacemaker group compared to the transvenous pacemaker group (RR: 0.49, 95% CI: 0.43-0.57). No significant heterogeneity was reported among the study results.

Secondary Outcomes

The outcomes of secondary importance are presented in Table 3. A noteworthy observation is the notable disparity in the risk of certain complications between the leadless and transvenous pacemaker groups. Specifically, patients in the leadless group exhibited significantly reduced risks of pneumothorax and endocarditis compared to those in the transvenous group. Conversely, the risk of pericardial effusion was found to be substantially greater in the leadless pacemaker group compared to the alternative study group. No statistically significant differences were detected between the two groups concerning the risks of hemothorax and hematoma.

**Table 3:** Comparison of secondary outcome between leadless and transvenous pacemakers RR: Risk ratio; CI: Confidence interval RR>1 signifies risk of outcome is greater in leadless group, while RR<1 signifies risk of outcome is greater in transvenous group

Outcome	RR	95% CI	I-square
Pneumothorax	0.22	0.07-0.71	17%
Pericardial effusion	2.39	1.37-4.17	0%
Endocarditis	0.18	0.06-0.55	0%
Required reintervention	0.55	0.47-0.66	0%
Hemothorax	0.94	0.65-1.36	0%
Hematoma	0.58	0.21-1.66	0%

Meta-regression

Table 4 shows the results of meta-regression taking total complications as the dependent variable. Hypertension is statistically significant in predicting total complications (p = 0.021). The coefficient's value further suggests a positive relationship between hypertension and total complications. No significant impact of diabetes, heart failure history, gender, and age is there in predicting total complications.

**Table 4:** Meta-regression taking total complications as a dependent variable The statistical test used to assess the significance of the relationships between predictor variables and the total complications is the Wald Test.

Factor	Coefficient	P-value
Diabetes	0.000085	0.213
Gender	-0.000033	0.089
Hypertension	0.000079	0.021
Heart failure	-0.001271	0.307
Age	0.00011	0.161

Discussion

In this meta-analysis, we compared the safety of a transvenous pacemaker with a leadless pacemaker. Patients with leadless pacemakers had a lower risk of total complications, device-related complications, pneumothorax, and endocarditis. The risk of reintervention was significantly lower in the leadless pacemaker group. However, compared to a transvenous pacemaker, the risk of pericardial effusion was significantly higher in the leadless pacemaker group. The findings of this meta-analysis confirm the value of leadless pacemakers, which is in line with ESC guidelines [26]. The findings of this meta-analysis are also in line with previous meta-analyses comparing the safety of leadless pacemakers and transvenous pacemakers [27]. Transvenous pacemakers are equipped with leads and necessitate a pocket for housing the pacemaker generator. This characteristic renders them susceptible to complications such as pocket hematoma and pocket infection [7].

Comparatively, a higher incidence of endocarditis was observed in cases involving transvenous pacemakers in contrast to leadless pacemakers, often necessitating the removal of the lead [3] [24]. The absence of implanted leads could confer benefits in mitigating the risk of bloodstream infection and endocarditis, given their reduced potential for biofilm accumulation, thrombus formation, disturbances in flow dynamics, and interference with heart valves. Unlike their transvenous counterparts, leadless pacemakers eliminate the need for a subcutaneous pocket; their smaller surface area and intracardiac placement offer inherent advantages in terms of infection and endocarditis prevention [3].

In the context of this comprehensive review, it is evident that the risk of pericardial effusion tends to be higher in leadless pacemakers when compared to their transvenous counterparts. Out of the eight studies encompassing this particular outcome, five studies documented a notably greater incidence of pericardial effusion in patients with leadless pacemakers. It is important to note, however, that statistical significance in this regard was observed in only one of these studies [13]. Pooling the data from these studies, the collective incidence of pericardial effusion within the leadless pacemaker group was found to be 1.12%, in contrast to the 0.45% incidence observed in the transvenous pacemaker group. While these figures suggest a disparity, it is worth considering that the absolute difference in incidence is not substantially pronounced. Therefore, to draw more conclusive insights into this outcome, additional comprehensive studies are warranted. Given the novelty of leadless pacemaker implantation, it is plausible that a learning curve associated with this relatively new technology might contribute to the observed increase in cardiac injury, including pericardial effusion. The process of implanting leadless pacemakers likely demands a certain level of procedural mastery that could influence complication rates. Furthermore, it is conceivable that local irritation stemming from the tines that anchor the leadless pacemaker to the myocardium could potentially lead to pericardial inflammation, thus contributing to the observed trend of increased pericardial effusion [28].

A crucial aspect that will shed further light on this matter is the long-term follow-up of patients. The leadless pacemaker offers advantages over transvenous pacemakers due to its reduced complications and enhanced safety and effectiveness. However, previous clinical studies on transvenous lead placements have shown that placing the pacemaker in the mid-septum, as opposed to the apex, significantly reduces the risk of pericardial tamponade and myocardial perforation. Similarly, research involving the midseptal implantation of the Micra leadless pacemaker demonstrates increased safety, a narrower paced QRS duration, and decreased susceptibility to cardiac perforation compared to placing it in the right ventricular apex [29]. The mid-septum of the right ventricle is an ideal location for Micra leadless pacemakers due to its lower risk of myocardial perforation, attributed to the presence of more myocardial trabeculae and columnar carneae compared to the right ventricular apex and outflow tract [30]. This holds particularly true at the center of the septum. To enhance the precision of implantation, a study involving the ventriculography-guided deployment of Micra pacemakers within the septum was conducted. This technique proved to be significantly more accurate (93.8%) than the standard method of implantation (48.1%). Additionally, while maintaining adequate pacing parameters, the use of right ventriculography substantially reduced fluoroscopy exposure time and radiation dose [29].

In this study, we merged data from current comparative research to assess dissimilarities in complications between conventional pacemakers and leadless pacemakers. Our analysis demonstrated that leadless pacemakers exhibit an edge in terms of complications related to leads and the pocket site. However, regarding other complications, disagreements between leadless pacemakers and traditional pacemakers persist. Concurrently, it is important to acknowledge that due to the absence of extensive follow-up data for leadless pacemakers, our grasp of their long-term safety concerning complications remains undisclosed. This underscores a significant knowledge gap concerning prolonged complications associated with these modern devices.

The present meta-analysis has certain limitations. All included studies were observational in nature and therefore confounding variables and other biases could not be excluded. Secondly, most of the included studies used Micra as the leadless pacemaker and therefore we cannot extrapolate these findings to AVEIR leadless pacemakers. Lastly, due to a lack of patient-level data, we were not able to perform subgroup analysis. In the future, it is essential to conduct additional studies to gain a deeper understanding of the populations that will benefit most from leadless and transvenous pacemakers.

## Conclusions

In conclusion, this meta-analysis offers valuable insights into the safety profiles of leadless pacemakers compared to transvenous pacemakers. The endpoint analysis strongly supports the notion that leadless pacemakers present a lower risk of total complications, device-related complications, pneumothorax, and endocarditis when compared to transvenous pacemakers. Additionally, the observed reduction in the risk of reintervention within the leadless pacemaker group underscores its potential clinical benefits. It is important to acknowledge the limitations arising from the lack of extensive long-term follow-up data for leadless pacemakers. As technology evolves, continued research will be essential in uncovering the full spectrum of prolonged complications associated with these devices.
